# The Protein Kinase C Inhibitor Enzastaurin Exhibits Antitumor Activity against Uveal Melanoma

**DOI:** 10.1371/journal.pone.0029622

**Published:** 2012-01-12

**Authors:** Xinqi Wu, Meijun Zhu, Jonathan A. Fletcher, Anita Giobbie-Hurder, F. Stephen Hodi

**Affiliations:** 1 Department of Medical Oncology, Dana-Farber Cancer Institute and Harvard Medical School, Boston, Massachusetts, United States of America; 2 Melanoma Program, Dana-Farber/Brigham and Women's Cancer Center, Boston, Massachusetts, United States of America; 3 Department of Pathology, Brigham and Women's Hospital and Harvard Medical School, Boston, Massachusetts, United States of America; 4 Department of Biostatistics and Computational Biology, Dana-Farber Cancer Institute, Boston, Massachusetts, United States of America; Technische Universität München, Germany

## Abstract

GNAQ mutations at codon 209 have been recently identified in approximately 50% of uveal melanomas (UM) and are reported to be oncogenic through activating the MAPK/Erk1/2 pathway. Protein kinase C (PKC) is a component of signaling from GNAQ to Erk1/2. Inhibition of PKC might regulate GNAQ mutation-induced Erk1/2 activation, resulting in growth inhibition of UM cells carrying GNAQ mutations. UM cells carrying wild type or mutant GNAQ were treated with the PKC inhibitor enzastaurin. Effects on proliferation, apoptosis, and signaling events were evaluated. Enzastaurin downregulated the expression of several PKC isoforms including PKCβII PKCθ, PKCε and/or their phosphorylation in GNAQ mutated cells. Downregulation of these PKC isoforms in GNAQ mutated cells by shRNA resulted in reduced viability. Enzastaurin exhibited greater antiproliferative effect on GNAQ mutant cells than wild type cells through induction of G1 arrest and apoptosis. Enzastaurin-induced G1 arrest was associated with inhibition of Erk1/2 phosphorylation, downregulation of cyclin D1, and accumulation of cyclin dependent kinase inhibitor p27^Kip1^. Furthermore, enzastaurin reduced the expression of antiapoptotic Bcl-2 and survivin in GNAQ mutant cells. Inhibition of Erk1/2 phosphorylation with a MEK specific inhibitor enhanced the sensitivity of GNAQ wild type cells to enzastaurin, accompanied by p27^Kip1^ accumulation and/or inhibition of enzastaurin-induced survivin and Bcl-2 upregulation. PKC inhibitors such as enzastaurin have activity against UM cells carrying GNAQ mutations through inhibition of the PKC/Erk1/2 pathway and induction of G1 arrest and apoptosis. Inhibition of the PKC pathway provides a basis for clinical investigation in patients with UM.

## Introduction

Ocular melanomas represent approximately 5% of all melanomas, with a majority of these being uveal in origin [1]. Uveal melanoma (UM) is the most common primary intraocular malignant tumor in adults, with an annual incidence of seven cases per million [2]. Approximately 50% of UM patients develop metastatic melanoma to the liver within 15 years of initial diagnosis. With distant metastases, there currently is no effective treatment modality. The median survival for UM patients with metastasis is less than six months [1].

The etiology of UM has not been fully understood. Although uveal and cutaneous melanomas arise from the same cell type, they have distinct genetic alterations. Genetic mutations in the TP53, *BRAF, RAS, CDKN2* and *PTEN* genes are common in cutaneous melanoma but rare in UM [3]. Drugs commonly used to treat cutaneous melanoma seldom produce durable responses in UM patients. The preponderance of liver metastases in uveal melanoma patients has focused therapeutic effort in local control of metastatic disease for palliation [4], [5]. Recently, somatic mutations in the GNAQ gene have been identified in about 50% of UM and 83% blue naevi [6], [7]. GNAQ mutations occurring at codon 209 of the RAS-like domain result in constitutive activation of the MAPK/Erk1/2 pathway in melanocytes and confer dominantly acting oncogenic functions to GNAQ [7]. The *GNAQ* gene encodes for the α subunit of q class of heterotrimeric GTP binding protein (Gq) that mediates signals from G-protein-coupled receptors (GPCRs) and stimulates all four isoforms of β phospholipase C (PLCβ) [8]. PLCβ enzymes catalyze the hydrolysis of phosphatidylinositol biphosphate, to release inositol trisphosphate and diacylglycerol (DAG) that function as second messengers and propagate and amplify the Gα-mediated signal through stimulation of protein kinase C (PKC). It has been hypothesized that signaling from GNAQ to MAPK/Erk1/2 is transmitted through DAG/PKC [9].

The PKC family is a widely expressed group of serine/threonine kinases comprising at least twelve isoforms [10]. PKCs are involved in key cellular processes including cell proliferation, apoptosis, and differentiation. Increased PKC expression and activity have been demonstrated in many cancers [10]–[13]. PKCs may play important roles in tumor formation and progression, invasiveness of cancer cells, and chemoresistance [13]–[15]. The mechanisms by which PKCs contribute to tumorigenesis, however, are not fully understood [16].

Enzastaurin (LY317615) is a potent and selective competitive inhibitor of PKCβ at low concentrations (IC50, 6 nmol/L) [17] and inhibits other PKC isoenzymes at higher concentrations [18]. In addition, enzastaurin targets the phosphatidylinositol 3-kinase/AKT pathway, and inhibits phosphorylation of GSK3β (Ser9) and ribosomal protein S6 (Ser240/244) [18]. Although enzastaurin was initially developed as an antiangiogenic agent, it also has direct proapoptotic and antiproliferative activities on various human cancer cells [18]–[25]. Therefore, enzastaurin may exhibit antitumor activity through multiple mechanisms affecting both tumor angiogenesis and apoptosis.

Given the importance of PKC in tumorigenesis [10], [15] and potentially in GNAQ mutation-induced MAPK activation [7], we hypothesized that PKC may provide new opportunities for therapeutic intervention of UM carrying GNAQ mutations. In the present study, we tested this hypothesis by examining the response of UM cells with wild type or mutant GNAQ toward the antiproliferative and proapoptotic action of enzastaurin and characterized the underlying signaling and molecular mechanisms.

## Results

### UM cells harboring GNAQ mutations experience increased inhibition of cell viability by enzastaurin

**Figure 1:**
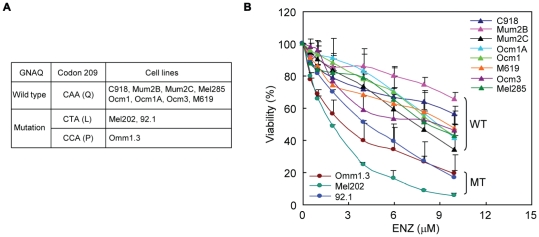
UM cells that harbor GNAQ mutations are more sensitive to the anitproliferative activity of enzastaurin. A, UM cell lines used in the present study. B, enzastaurin exhibited greater antiproliferative effects on UM cell lines carrying GNAQ mutations (MT) than those with wild type GNAQ (WT). Cells were treated with varying amount of enzastaurin for 72 hours and subjected to MTS assay. Results are presented as mean ± SD of percent viability from three independent experiments.

A panel of eleven UM cell lines was used to evaluate the antiproliferative effect of enzastaurin (Figure 1A). DNA sequencing confirmed that cell lines Omm1.3 (accession number JN184781), Mel202 (accession number JN184779) and 92.1 (accession number JN184780) carry a mutation at codon 209 of GNAQ while the remaining cell lines are wild type for GNAQ. No mutations were found at codon 183 of GNAQ and at codons 209 and 183 of GNA11 in all these cell lines. Cell lines Ocm1 and Ocm3 have been reported to harbor BRAF V600E mutation [26]. While a dose-dependent decrease in viability was seen in all eleven UM cell lines tested, greater inhibition was noted in the three cell lines harboring GNAQ mutations (Figure 1B). The IC50 of enzastaurin was 2–4 µM for the cell lines with mutations, compared to 8 µM or greater for the wild type cell lines.

We next looked for evidence that the response of UM cells to enzastaurin is related to mutational status using statistical analyses. The statistical model results indicate that the effect of enzastaurin upon viability depends upon both the mutational status and enzastaurin concentration (interaction p-value = 0.0001). On average, viability was reduced less in the wild type cell lines, even at high concentrations of enzastaurin (Fig. S1). For wild type cell lines, viability decreased by approximately 7% (95% CI: 6.6% to 8.2%) for each unit increase in enzastaurin concentration. In mutated cell lines, there was an estimated decrease of 21% (95% CI: 19.8% to 22.6%) in viability for each unit increase in enzastaurin concentration (Table S1). Based upon the estimated equations, the EC50 values are 9.15 µM and 2.85 µM for wild type and mutated cell lines, respectively. There is little evidence for differences in response in enzastaurin concentrations of 1 µM or less. At concentrations of 2 µM or above, there was a statistically significant reduction in the viability of mutated cell lines compared with wild type.

### Enzastaurin induces cell cycle arrest and apoptosis in UM cells

**Figure 2:**
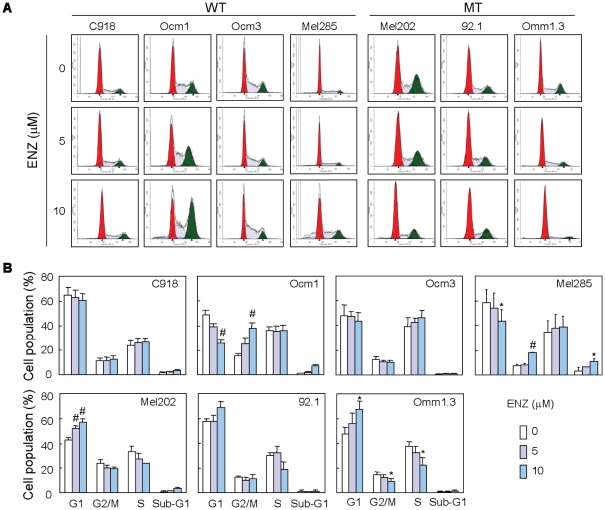
Enzastaurin induced G1 arrest in UM cells carrying GNAQ mutations. A, cells were treated with enzastaurin for 48 hours, stained with PI and subjected to FACS analysis of cell cycle distribution. B, percentages of G1, S, G2/M, and sub-G1 populations were estimated using software ModFit. Mean percentages of each population from 3–5 independent experiments are presented as bar graphs. * P<0.05, versus control; # P<0.01, versus control. WT = GNAQ wild type; MT = GNAQ mutation.

**Figure 3:**
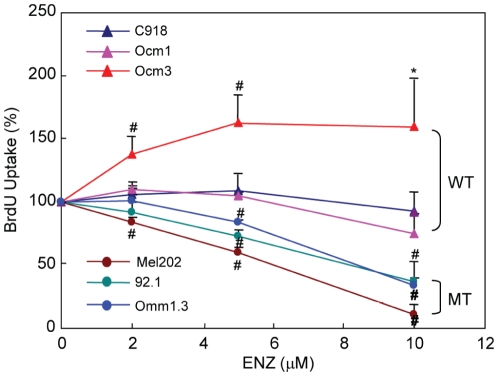
Effect of enzastaurin on BrdU incorporation. UM cells were treated with enzastaurin at indicated concentrations for 40 h and then labeled with BrdU for additional 6–8 hours. BrdU incorporation was presented as % of untreated control. Mean ± SD of 3 independent experiments are shown. * P<0.05, versus control; # P<0.01, versus control. WT = GNAQ wild type; MT = GNAQ mutation.

To better understand the differential responses of UM cells based on GNAQ mutational status, we investigated cell cycle progression alterations with drug exposure. Enzastaurin treatment for 48 hours significantly increased the G1 population while decreasing the S population in all three cell lines harboring GNAQ mutations (Figure 2). In agreement with these findings, enzastaurin significantly decreased BrdU incorporation in mutant cell lines (Figure 3). These results suggest that enzastaurin induced G1 arrest in the cell lines harboring mutations. In comparison, the G1 population of the wild type cell lines was either unaltered (C918 and Ocm3) or decreased by enzastaurin (Ocm1 and Mel285). A significant increase in the G2/M population was observed in Ocm1 and Mel285 cells. A mild increase in the S population and a significant increase in BrdU uptake were observed in Ocm3 cells treated with 5 and 10 µM enzastaurin (Figures 2 and 3).

**Figure 4:**
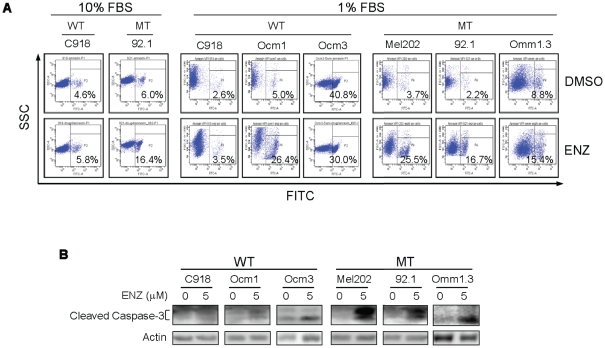
Enzastaurin induced apoptosis in UM cells expressing mutant GNAQ. A, cells were treated with 4 and 5 µM enzastaurin in the presence of 1% and 10% FBS for 72 hours, respectively. Apoptotic cells were detected using Annexin V-FITC staining and FACS analysis. B, immunoblot analysis of cleaved casapase-3 in cells treated with 5 µM enzastaurin in the presence of 1% FBS for 48 hours. WT = GNAQ wild type; MT = GNAQ mutation.

As enzastaurin is known to induce apoptosis in many types of cancer cells [18]–[25], we next examined whether enzastaurin induced apoptosis of UM cells using Annexin V-FITC staining. Treatment with 4 µM enzastaurin for 72 hours induced a slight increase (10.4%) in apoptosis in mutant cell line 92.1 but not in the wild type cell line C918 (Figure 4, left panel). Because enzastaurin is highly bound by serum protein, we tested if reduced serum concentrations would increase its apoptotic effects. In the presence of 1% serum, treatment with 5 µM enzastaurin for 72 hours induced substantial apoptosis in the cell lines Mel202, 92.1 and Omm1.3 harboring GNAQ mutations, and in the wild type cell lines Ocm1 (harboring a BRAF mutation), but failed to do so in cell line C918 which is wild type for GNAQ (Figure 4A, right panel). An increase in cleaved caspase-3 fragments was also observed in enzastaurin-treated Mel202, 92.1 and Omm1.3 mutant cells and Ocm1 wild type cells, but not C918 cells (Figure 4B). These findings suggest that UM cells carrying GNAQ mutations and some GNAQ wild type/BRAF mutant cells are more sensitive to the apoptotic activity of enzastaurin and that enzastaurin exerted increased antiproliferative effect on GNAQ mutant UM cells through induction of G1 arrest and apoptosis.

#### Enzastaurin increases p27^Kip1^ expression and decreases the expression of cyclin D1 in GNAQ mutant UM cells

**Figure 5:**
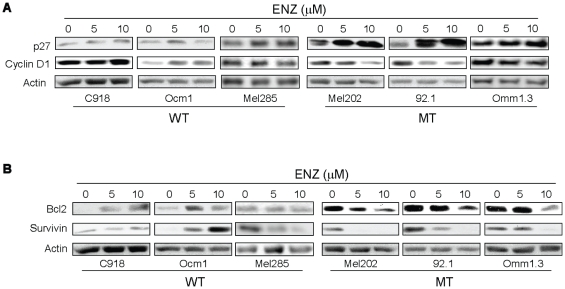
Effects of enzastaurin on the expression of cell cycle (A) and apoptosis (B) regulatory proteins in GNAQ wild type and mutant UM cells. Cells were treated with enzastaurin for 72 hours and analyzed by immunoblot analysis. WT = GNAQ wild type; MT = GNAQ mutation.

To understand the molecular mechanisms underlying enzastaurin-induced G1 arrest in GNAQ mutant UM cells, we investigated the enzastaurin response of cell cycle regulatory molecules, including cyclin D1 and p27^Kip1^ that regulate cell cycle progression through G1 phase [28]. In all three GNAQ mutant UM cell lines treated with enzastaurin, the expression of p27^Kip1^ was markedly increased, while the expression of cylin D1 was reduced (Figure 5A). Enzastaurin had minimal effect on p27 and cyclin D1 expression in GNAQ wild type cells. These findings are in line with enzastaurin-induced G1 arrest occurring only in GNAQ mutant lines, and suggest that cyclin D1 and p27 dysregulation are molecular mechanisms for enzastaurin-induced G1 arrest.

#### Enzastaurin decreases the expression of Bcl-2 and survivin in GNAQ mutant UM cells

We next examined the effect of enzastaurin on the expression of antiapoptotic (survivin, Bcl-2, Bcl-xL, XIAP, Mcl-1, and ARC) and proapoptotic (p53 and Bim) proteins. Enzastaurin significantly inhibited survivin expression in all three cell lines with GNAQ mutations and in GNAQ wild type cell line Mel285 (Figure 5B). It is noteworthy that the mutant cell lines expressed significantly higher levels of Bcl-2 compared with wild type cell lines (Figure 5B and unpublished data). Enzasaturin decreased Bcl-2 expression in the mutant cell lines and appeared to slightly increase (C918 and Ocm1 cells) or have little effect (Mel285) on its expression in the wild type cells. The expression of p53, Bcl-xL, XIAP, Mcl-1, Bim and ARC were not significantly altered by enzastaurin in either wild type or mutant cell lines (Figure S2).

#### Enzastaurin decreases Erk1/2 phosphorylation in UM cells harboring GNAQ mutations

**Figure 6:**
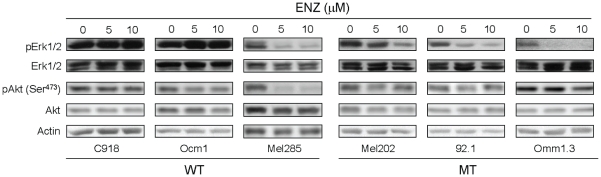
Effects of enzastaurin on Akt and Erk1/2 activation in UM cells. Cells were treated with enzastaurin for 72 hours and analyzed for the expression of Akt and Erk1/2 and their phosphorylation by immunoblot analysis. WT = GNAQ wild type; MT = GNAQ mutation.

We next investigated whether enzasaturin affected Akt and Erk1/2 activation that are critical for UM development. Erk1/2 activation has been reported to be induced by mutant GNAQ and has been found in UM independent of GNAQ, BRAF and RAS mutational status [7], [27], [34]. Akt but not Erk1/2 phosphorylation has been reported to be inhibited by enzastaurin in several types of cancer cells [18], [22], [24], [25], [35]–[39]. Interestingly, Erk1/2 phosphorylation was significantly suppressed in all three GNAQ mutant cell lines by enzastaurin but only in one GNAQ wild type cell line (Mel285) (Figure 6). In contrast, Akt (Ser473) phosphorylation was affected by enzastaurin only in GNAQ wild type cell line Mel285. Total Akt and Erk1/2 levels were not significantly affected by enzastaurin in the wild type as well as the mutant cell lines. These findings suggest that enzastaurin may exert its antiproliferative action on GNAQ mutant cells in part through targeting the MAPK pathway. Enzastaurin inhibited GSK3β phosphorylation and induced β-catenin expression in both wild type and mutant cell lines (Figure S3), consistent with its known mechanism of activity.

#### Inhibition of Erk1/2 phosphorylation increases the antiproliferation effects of enzastaurin on wild type UM cells

**Figure 7:**
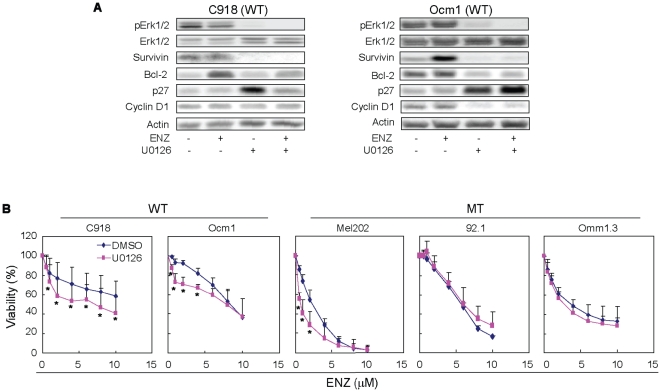
Effect of MEK inhibition on the antiproliferative action of enzastaurin. A, expression of Erk1/2, phospho-Erk1/2, survivin, Bcl-2, cyclin D1, and p27^Kip1^ in C918 and Ocm1 cells treated with enzastaurin in the absence or presence of U0126 (20 µM) for 72 hours. B, effect of U0126 on the antiproliferative activity of enzastaurin. Assay was performed as described in Figure 1 in the absence or presence of 10 µM U0126. Results are presented as mean ± SD of percent viability from 4–5 independent experiments. * P<0.05, versus DMSO at the same dose of enzastaurin.

To better understand the role of the MAPK pathway in the response of UM cells to enzastaurin, we treated C918 and Ocm1 cells with enzastaurin in the presence of 20 µM U0126, an MEK specific inhibitor. As expected, U0126 inhibited Erk1/2 phosphorylation, but had little effect on total Erk1/2 (Figure 7A). U0126 reduced both basal and enzastaurin-induced expression of survivin in C918 and Ocm1 cells (Figure 7A). U0126 reduced basal expression of Bcl-2 in Ocm1 cells and enzastaurin-induced expression of Bcl-2 in C918 and Ocm1 cells. Furthermore, U0126 increased p27^Kip1^ expression in Ocm1 and C918 cells which was further augmented in the presence of enzastaurin in Ocm1 cells but not in C918 cells (Figure 7A). The expression of cyclin D1 was repressed in Ocm1 cells treated with U0126 alone or in combination with enzastaurin (Figure 7A). Interestingly, U0126 increased the antiproliferative effects of enzastaurin on C918 cells, resulting in an IC50 comparable to that found in cells harboring GNAQ mutations (Figure 7B). U0126 also enhanced the antiproliferation effects of enzastaurin on Ocm1 cells at lower concentrations (1–4 µM versus 6–10 µM) (Figure 7B). Similar effect on proliferation was also observed in enzastaurin-treated C918 and Ocm1 cells with another MEK inhibitor AZD6244 (Figure S4). As UM cells harboring GNAQ mutations concerned, only Mel202 cells showed increased sensitivity to enzastaurin in the presence of U0126 (Figure 7B). U0126 and AZD6244 alone showed varying inhibitory effects on proliferation of GNAQ wild type and mutant UM cell lines, with AZD6244 having greater inhibitory effects (Figure S5).

#### Enzastaurin affects the expression and phosphorylation of PKCβ, PKCε and PKCθ in UM cells harboring GNAQ mutations

**Figure 8:**
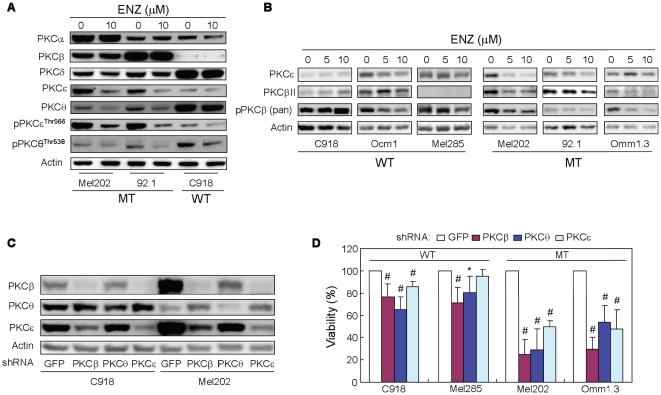
PKCβ, PKCε and PKCθ are functionally important for UM cells harboring GNAQ mutations. A and B, enzsaurin reduced the expression and phosphorylation of PKCβ, PKCε and PKCθ. Cells were treated with enzastaurin for 6 hours in the absence of serum (A) or for 72 hours in the presence of 10% FBS (B). pPKCε Thr566 was detected using pan p-PKC antibody raised against pPKCζ Thr410. Note that the expression of PKCβ was under detectable level of Western blot in Mel285 cells (B). C, knockdown of PKCβ, PKCε, and PKCθ by shRNA. C918 and Mel202 cells were infected with lentivurs expressing shRNA for each PKC isoform for 4 days and protein levels of these isoforms were determined by immunoblot blot analysis. D. Effects of knockdown of PKCβ, PKCε, and PKCθ on viability of UM cells. Cell viability was determined 4 days after infection with lentivrus expressing PKC shRNA using MTS assay. Results are presented as mean ± SD of percent viability from 3 independent experiments. *P<0.05 and # P<0.01, versus cells expressing GFP control shRNA.

In order to determine whether the antiproliferative action of enzastaurin is mediated via inhibition of PKC, we investigated the effect of enzastaurin on the expression and activation of PKC isoforms. Immunoblotting revealed varying expression of PKC isoforms in UM cell lines (Figure 8A). UM cell treatment with enzastaurin in the absence of serum for 6 hours inhibited the expression of PKCθ and PKCε in Mel202 and 92.1 cells, but not in C918 cells (Figure 8A). By contrast, enzastaurin had little effect on PKCα, PKCβ, and PKCδ expression. Furthermore, enzastaurin inhibited PKCε phosphorylation dramatically in Mel202 and 92.1, but not C918 cells, whereas PKCθ phosphorylation was partially inhibited in all (Figure 8A).

It has been reported that enzastaurin downregulates the expression of PKC isoforms [24], [29], and we therefore examined the effect of enzastaurin on the expression of PKCβII, PKCε, and phospho-PKCβ in UM cells after prolonged (72 hours) treatment and in the presence of serum (Figure 8B). As described above, enzastaurin decreased the expression of PKCε in 92.1 and Mel202 cells but not in C918 and Mel285 cells. Enzastaurin decreased PKCε expression in Ocm1 cells but not in Omm1.3 cells, indicating its effect on PKCε expression is independent of GNAQ status. Similarly, varying levels of PKCβII expression were seen among these UM cell lines. While enzastaurin did not significantly alter PKCβII expression in C918 and Ocm1 cells, it did decrease PKCβII expression in Omm1.3, Mel202, and 92.1 cells harboring GNAQ mutations. Enzastaurin decreased PKCβ phosphorylation in Ocm1, Mel285, Omm1.3, Mel202, and 92.1 cells, with the most profound change seen in Omm1.3 cells. In comparison, PKCβ phosphorylation was slightly increased in C918 cells after treatment with 10 µM enzastaurin.

#### PKCθ, PKCβ, and PKCε are functionally important for UM cells harboring GNAQ mutations

As enzastaurin inhibited the expression and/or phosphorylation of PKCβ, PKCε, and PKCθ in UM cells harboring GNAQ mutations, we investigated the functional importance of these PKC isoforms by shRNA-mediated downregulation. Infection of C918 and Mel202 cells with lentivirus expressing shRNA of PKCβ, PKCε or PKCθ reduced the protein levels of corresponding isoforms in these cells (Figure 8C). It is noteworthy that in both C918 and Mel202 cells expressing PKCβ shRNA, PKCε protein levels were also reduced. A reduction in PKCθ protein abundance was also detected in PKCβ shRNA expressing Mel202 cells. In C918 and Mel202 cells expressing PKCε, PKCβ expression was downregulated as well. In Mel202 cells expressing PKCθ, the expression of PKCβ and PKCε was modestly increased. Notably, expression of PKCβ, PKCθ or PKCε shRNA significantly decreased viability of Mel202 and Omm1.3 cells (Figure 8D). A reduction in viability was also seen in C918 cells expressing shRNA of PKCβ, PKCε or PKCθ and in Mel285 cells expressing PKCβ or PKCθ, but to less extent compared to GNAQ mutated cells. Significant viability reduction was seen with second shRNA for each of these PKC isoforms in Mel202 cells but not in C918 cells (Figure S6). These findings suggest that PKCθ PKCβ, and PKCε may functionally be more important for UM cells harboring mutated than those with wild type GNAQ. In supporting this notion, we were able to establish C918 but not Mel202 stable cell line expressing PKCθ shRNA (data not shown). Altogether our findings support the notion that enzastaurin may exert anti-proliferative action via the inhibition of these PKC isoforms.

## Discussion

GNAQ mutations at codon 209 have been recently found in nearly 50% of UM patients [6], [7]. These mutations can lead to activation of a number of cell signaling pathways. In the present study, we demonstrate for the first time that UM cell lines harboring GNAQ mutations are more sensitive to the antiproliferative effects of the PKC inhibitor enzastaurin than those possessing wild type GNAQ. Enzastaurin inhibits proliferation of mutant UM cells through induction of G1 cell cycle arrest and apoptosis. We have further characterized signaling and molecular mechanisms underlying differential responses of GNAQ wild type and mutant cells to enzastaurin.

The PI3K/Akt and MAPK pathways are frequently activated in malignant tumors [30]–[32]. Erk1/2 activation is commonly found in UM, independent of GNAQ, RAS, and BRAF mutational status [27], [33], [34], and are crucial for UM development [34]. GNAQ mutations have been reported to be oncogenic through activating the Erk1/2 pathway in UM cells [7]. In the current study, we show that enzastaurin reduced Erk1/2 phosphrylation in all three GNAQ mutant UM cell lines and in one wild type cell line (Mel285). Erk1/2 phosphorylation has been shown to be unaltered or increased by enzastaurin in several cancer types [22], [24], [35]–[39], whereas Akt phosphorylation has been reported to be downregulated by enzastaurin, likely through an indirect mechanism as Akt is not a direct target of the drug [18], [24], [25]. However, enzastaurin has also been reported to have little effect on Akt phosphorylation in glioma cells [36]. In the UM cells studied here, Akt phosphorylation was only affected in Mel285 cells by enzastaurin. Interestingly, although both Akt and Erk1/2 phosphorylation were decreased by enzastaurin, Mel285 cells, like other GNAQ wild type cells, were less sensitive to enzastaurin in comparison to GNAQ mutated cells where only Erk1/2 phosphorylation was affected. In agreement with sensitivity to enzastaurin, inhibition of Erk1/2 phosphorylation was accompanied by increased p27^Kip1^ accumulation and decreased expression of cyclin D1, Bcl-2 and survivin in GNAQ mutant cells whereas only survivin was downregulated in Mel285 cells. Furthermore, inhibition of Erk1/2 phosphorylation by MEK1/2 inhibitors increased sensitivity of GNAQ wild type cells to enzastaurin and was associated with similar alterations in the expression of p27^Kip1^, cyclin D1, Bcl-2 and/or survivin to GNAQ mutant cells treated with enzastaurin. Our findings suggest that the suppression of Erk1/2 phosphorylation may be the major contributor to the increased sensitivity of GNAQ mutant UM cells to the antiproliferative action of enzastaurin through altering the expression of p27, ccyclin D1, Bcl-2 and survivn. These observations further support the oncogenic role for GNAQ mutations via activation of MAPK [7].

The signaling pathways downstream of GNAQ are multifold and include activation of the PKC family members [8]. Our results indicate that UM cell lines have varying expression and phosphorylation patterns of PKC isoforms, independent of GNAQ mutational status. The effects of enzastaurin on the expression and phosphorylation of PKC isoforms in UM cells are complex. Additional studies are needed to determine whether GNAQ mutational status influences the effects of enzastaurin on various PKC isoforms and the potential therapeutic ramifications of these effects. Nonetheless, some PKC isoforms were downregulated by enzastaurin in UM cell carrying GNAQ mutations. In particular, the expression and phosphorylation of PKCθ, PKCε, and PKCβ were reduced by enzastaurin in GNAQ mutated cells. Our functional studies revealed that these PKC isoforms are indeed more critical for growth of UM cells harboring GNAQ mutations than those with wild type GNAQ. Together, our findings suggest that enzastaurin may exert increased antiproliferative action through inhibiting these PKC isoforms in GNAQ mutated UM cells. Inhibition of these isoforms may play a role in enzastaurin-induced inhibition of Erk1/2 phosphorylation, since activation of PKCε and PKCβII have been shown to trigger several major signaling pathways including MAPK [40], [41]. In addition, the inhibition of PKCβII by enzastaurin or small interfering RNA decreased Erk1/2 phosphorylation in metastatic hepatocellular carcinoma cells [42]. It is noteworthy that although enzastaurin had little effect in general on the expression and/or phosphorylation of PKC isoforms in GNAQ wild type C918 cells, it did decrease the expression of PKCε and PKCβ phosphorylation in another GNAQ wild type cell line Ocm1. However, enzastaurin did not significantly alter Erk1/2 phosphorylation in both cell lines, suggesting other PKC isoforms and/or PKC independent mechanisms for Erk1/2 activation in Ocm1 cells. Complicating this interpretation, Ocm1 cells have been shown to carry the common V600E BRAF mutation that constitutively activates the MAPK pathway [43], [44]. Furthermore, PKCα and PKCδ have been reported to activate Erk1/2 in mouse melanoma [51]. Both PKCα and PKCδ are expressed in Ocm1 cells (unpublished data).

In the present study, we demonstrate that enzastaurin-induced antiproliferation of UM cells carrying GNAQ mutations is associated with G1 arrest. Enzastaurin has been shown to have little effect on cell cycle progression in many types of cancers [25]. Recently, it was reported to induce G1 arrest in non-small cell lung cancer cells [37]. Enzastaurin-induced G1 arrest in UM cells is associated with downregulation of the positive cell cycle regulators cyclin D1 and upregulation of negative cell cycle regulator p27^Kip1^. This recapitulates the Erk1/2 inhibition-induced G1 arrest by MEK inhibition that is characterized by decreased expression of cyclin D1 and accumulation of p27^Kip1^ [45]–[47]. This further supports that enzastaurin may induce G1 arrest primarily through the MAPK pathway. Downregulation of survivin has been shown to be associated with rapamycin-induced G1 arrest [52] and may also play a role in enzastaurin-induced G1 arrest reported here. However, enzastaurin did not induce G1 arrest in Mel285 cells where survivin expression was suppressed. This suggests that survivin downregulation alone is not sufficient to induce G1 arrest and alterations in other G1-S transition regulatory proteins such as p27 and cyclin D1 are required. G1 arrest can be induced by reduced expression of other cell cycle regulatory proteins including cylins D3, E and A and CDK4 [52], [53]. It remains to be examined whether enzastaurin downregulates the expression of these proteins. Enzastaurin either has no effect on cell cycle or increases G2/M population in UM cell lines with wild type GNAQ. It is intriguing that one of the GNAQ wild type UM cell lines was found to harbor a BRAF mutation and the implication of this biology to a PKC inhibitor such as enzastaurin remains to be investigated.

We have also demonstrated that enzastaurin induces apoptosis in UM cells carrying GNAQ mutation. Enzastaurin-induced apoptosis is associated with the downregulation of antiapoptotic Bcl-2 and survivin, while the expression of other common apoptosis regulators was not significantly altered. Both MAPK and Akt pathways have been reported to induce Bcl-2 and survivin expression [48]–[50]. As the Akt pathway was minimally affected by enzastaurin, the downregulation of Bcl-2 and survivin by enzastaurin may be the result of decreased activation of the MAPK pathway in cells carrying GNAQ mutations. This is supported by our findings that MEK inhibition also downregulated the expression of Bcl-2 and/or survivin in the wild type cells. Interestingly, the expression of survivin but not Bcl-2 was decreased in Mel285 cells where both Erk1/2 and Akt phosphorylation was suppressed by enzastaurin. This suggests that additional signaling pathway(s) may be involved in Bcl-2 expression in these cells. The molecular mechanisms underlying apoptosis induced by enzastaurin seen in some UM cell lines with wild type GNAQ remains to be investigated.

In summary, compared with UM cells with wild type GNAQ, the PKC inhibitor enzastaurin at low micromolar concentrations exerts significant antiproliferative effect on UM cells carrying GNAQ mutations through targeting PKC/MAPK pathways with induction of G1 arrest and apoptosis. Our findings suggest that enzastaurin and other compounds affecting PKC and related pathways could be of therapeutic potential for UM.

## Materials and Methods

### Cell Lines

UM cell lines M619, C918, Mum2B, Mum2C, Ocm1 and Ocm3 were kindly provided Dr. L. Chin (Dana-Farber Cancer Institute, Boston, MA). UM cell lines Mel202, 92.1 and Ocm1A were kindly provided by Dr. J. W. Harbour (Washington University, St. Louis, MI), Mel285 by Dr. D. Fisher (Massachusetts General Hospital, Boston, MA), and Omm1.3 by Dr. B. Bastian (University of California, San Francisco, CA). It has been reported by Folberg and colleagues in studies authenticating cell lines that Ocm1 and Mum2C are from the same patient, and that M619, C918, and Mum2B are from the same patient [26]. UM cells were cultured in RPMI 1640 supplemented with 10% FBS, 50 µg/ml penicillin, and 100 µg/ml streptomycin at 37°C and 5% CO_2_. In some experiments, in order to analyze the effect of enzastaurin on PKC isoforms, cells were incubated with or without enzastaurin in serum-free media.

### Cell viability assay

Cells were seeded in 96-well plates at 2×10^3^ cells per well and incubated overnight. Cells were then treated with enzastaurin (provided by Eli Lily and Company, Indianapolis, Indiana), MEK1/2 inhibitor U0126 (Cell Signaling Technology, Danvers, MA) or AZD6244 (Selleck Chemicals, Houston, TX) for three days. Cell viability was determined by MTS proliferation assay (Promega, Madison, WI) as per manufacturer's instructions. For signaling pathway experiments, cells were treated with enzastaurin in the presence of U0126 (10 µM) or AZD6244 (2 µM).

### Cell cycle analysis

Cells were harvested by trypsinization and fixed in cold ethanol. After treatment with RNase, cells were stained with PI and subjected to flow cytometry analysis (FACS) to determine cell cycle distribution.

### BrdU uptake analysis

Cells were plated in 96-well plates at 4×10^3^ cells/well in growth medium and incubated overnight. Cells were treated with 0, 5, and 10 µM enzastaurin for 40 h. BrdU were added to the cells and allowed the cells to be labeled for 6–8 hours. Cell labeling with BrdU, fixation and detection were performed using a BrdU Cell Proliferation Assay kit as guided by the manufacturer (EMD Chemicals, Gibbstown, NJ). BrdU incorporation was presented as % of untreated control.

### Analysis of apoptosis

Cells were stained with Annexin V-FITC using an Apoptosis detection kit (BD Biosciences, San Jose, CA) and subjected to flow cytometry analysis according to the instructions provided by the manufacturer. Compensation for enzastaurin autofluorescence was performed.

### Immunoblot analysis

Cells were treated in lysis buffer (Cell Signaling) supplemented with protease inhibitors (Roche, Minneapolis, MN). Equal amounts of cell lysates were subjected to electrophoresis on SDS polyacrylamide gels. Proteins were transferred onto nitrocellulose membranes (Bio-Rad Laboratories, Hercules, CA). The membranes were blocked with 5% nonfat dry milk or BSA, incubated with primary and second antibodies, and developed by ECL. Antibodies against PKC isoforms were: PKCθ (BD Biosciences #610090); PKCθ Thr538 (Cell Signaling #9377); PKCθ/δ Ser643/676 (Cell Signaling #9376); PKCε (BD Biosciences #610085); pan-phospho-PKC (PKCγ Thr514, Cell Signaling #9379, also recognizing PKCs α, βI, βII, ε, θ, δ, and η, when phosphorylated at a residue homologous to PKCγ Thr514); pan-phospho-PKC (PKCζ Thr410, Cell Signaling #2060, recognizing PKCs α, βI, βII, γ, ε, θ, δ, η, and ι, when phosphorylated at a residue homologous to PKCζ Thr410); and pan-phospho-PKCβII Ser660 (Cell Signaling #9371). Antibodies against Akt, phospho-Akt, Erk1/2, phospho-Erk1/2, cyclin D1, p53, β-catenin, Bcl-2, Bcl-xL, XIAP, survivin, GSK3β and phospho-GSK3β were purchased from Cell Signaling Technology. Antibodies against PKCβII, P27^Kip1^, ARC, and BIM were purchased from Santa Cruz Biotechnology (Santa Cruz, CA). Actin antibody was purchased from Sigma-Aldrich (St. Louis, MI). Data shown are representatives of 2 or more experiments.

### Knockdown of PKC isotypes by shRNA

The constructs (pLKO.1-puro) containing shRNA target sequences for PKCβ, PKCε, PKCθ or GFP (used as control) were provided by Dana-Farber Cancer Institute shRNA Core Facility. Lentivirus was produced by co-transfection of 293T cells with pLKO.1-puro, pCMVΔR8.91 and pMD.G according to the protocol provided by the facility. Cells were infected with virus for 4 days and cell viability was determined using MTS assay.

### DNA sequence data deposition

All new data has been deposited in GenBank (accession numbers JN184778, JN184779, JN184780 and JN184781).

### Statistical Methods

Graphs of the original viability data of UM cell lines suggested a curvilinear relationship between proliferation and concentration of enzastaurin. For comparison between GNAQ wild type and mutated groups, viability data were natural log (base *e*) transformed for the statistical models to make the concentration-response relationship more linear. Log-transformed data were fit using random effects, mixed models, with mutation status, concentration of enzastaurin, and their interaction as independent predictors. Maximum likelihood estimates from the model are summarized with 95% confidence intervals. Statistical analyses were conducted using SAS 9.2 (SAS Institute Inc., Cary, NC). For statistic analysis of changes in viability and cell cycle distributions of each cell line two-tailed *t*-test was used.

## Supporting Information

Figure S1 **Statistical modeling of data from viability assays.** A, the original data after log transformation. For each cell line, the replicates were averaged and represented as dots. B, transformed data were averaged across replicates by mutational status.
(TIF)

Figure S2 **Immunoblot analysis of apoptosis regulatory proteins in UM cells with or without enzastaurin treatment for 72 hours.**
(TIF)

Figure S3 **Effect of enzastaurin on GSK3β signaling in UM cells.** Cells were treated with enzastaurin for 72 hours in the presence of 10% FBS. Note that the expression of GSK3β and pGSK3β Ser9 was under detectable levels of immunoblot in Omm1.3 cells.
(TIF)

Figure S4 **Effect of AZD6244 on the antiproliferative activity of enzastaurin.** Assay was performed as described in Figure 1 in the absence or presence of 2 µM AZD6244. Results are presented as mean ± SD of percent viability from 2–3 independent experiments. * P<0.05, versus DMSO; # P<0.01, versus DMSO at the same dose of enzastaurin.
(TIF)

Figure S5 **Effect of MEK inhibitors on UM cell viability.** A, U0126. B, AZD6244. UM cells were treated with varying amount of U0126 or AZD6244 for 72 hours and subjected to MTS assay. Results are presented as mean ± SD of percent viability from 2 independent experiments.
(TIF)

Figure S6 **Effect of second shRNA for PKCβ, PKCε, and PKCθ on C918 and Mel202 cell viability. Experiments were performed as described in** **figure 8** **.** Results are presented as mean ± SD of percent viability from 3 independent experiments. # P<0.01 versus cells expressing GFP control shRNA.
(TIF)

Table S1 **Viability reduction rate of GNAQ mutant compared to wild type UM cell lines at various concentrations of enzastaurin.**
(TIF)
