# Supplementary figures and images for: The Protein Kinase C Inhibitor Enzastaurin Exhibits Antitumor Activity against Uveal Melanoma
Source: PLoS One. 2012 Jan 12;7(1):e29622. doi: 10.1371/journal.pone.0029622 (PMC3257235; doi:10.1371/journal.pone.0029622)

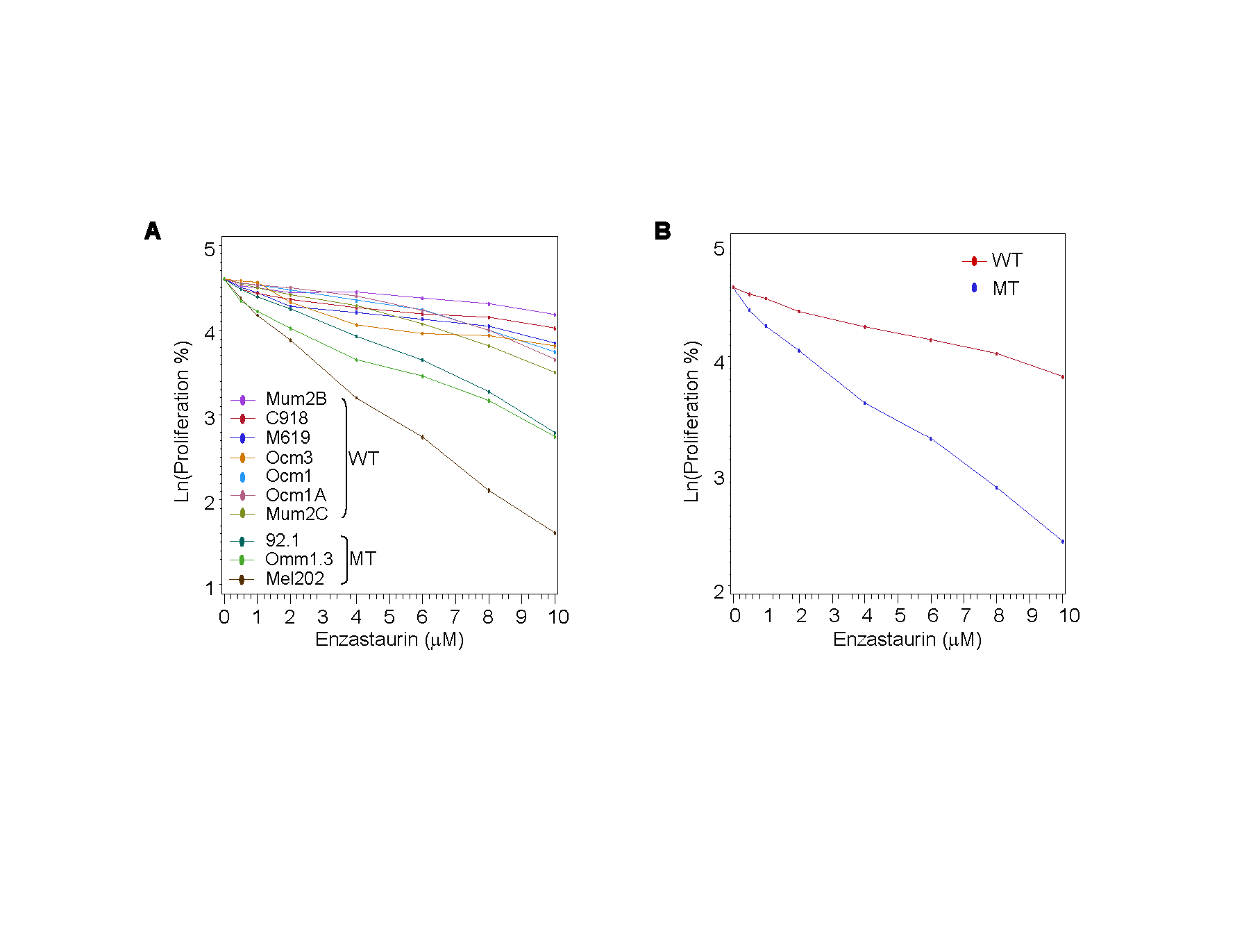

Supplement: Figure S1 — Statistical modeling of data from viability assays. A, the original data after log transformation. For each cell line, the replicates were averaged and represented as dots. B, transformed data were averaged across replicates by mutational status. (TIF) [file pone.0029622.s001.tif]

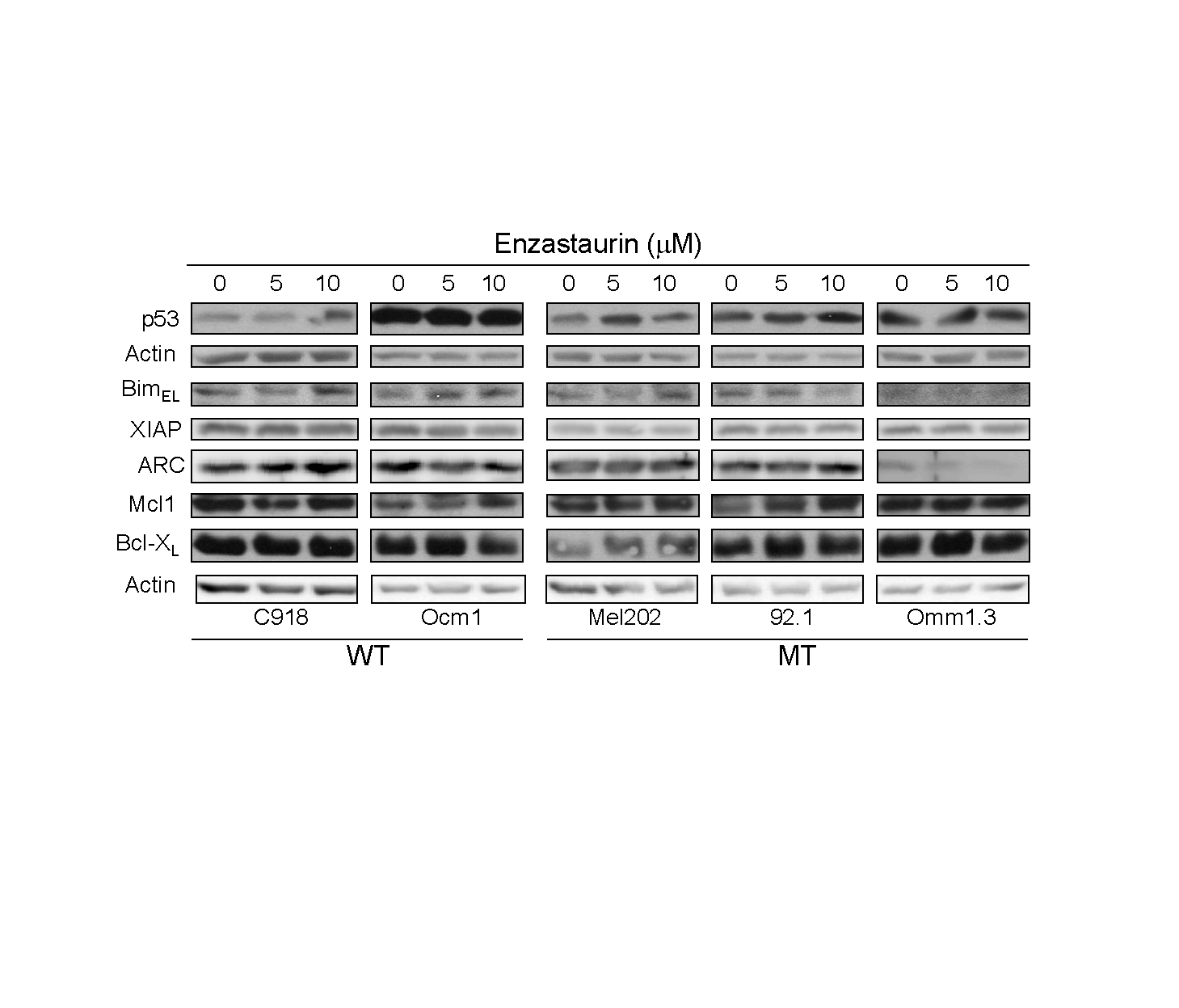

Supplement: Figure S2 — Immunoblot analysis of apoptosis regulatory proteins in UM cells with or without enzastaurin treatment for 72 hours. (TIF) [file pone.0029622.s002.tif]

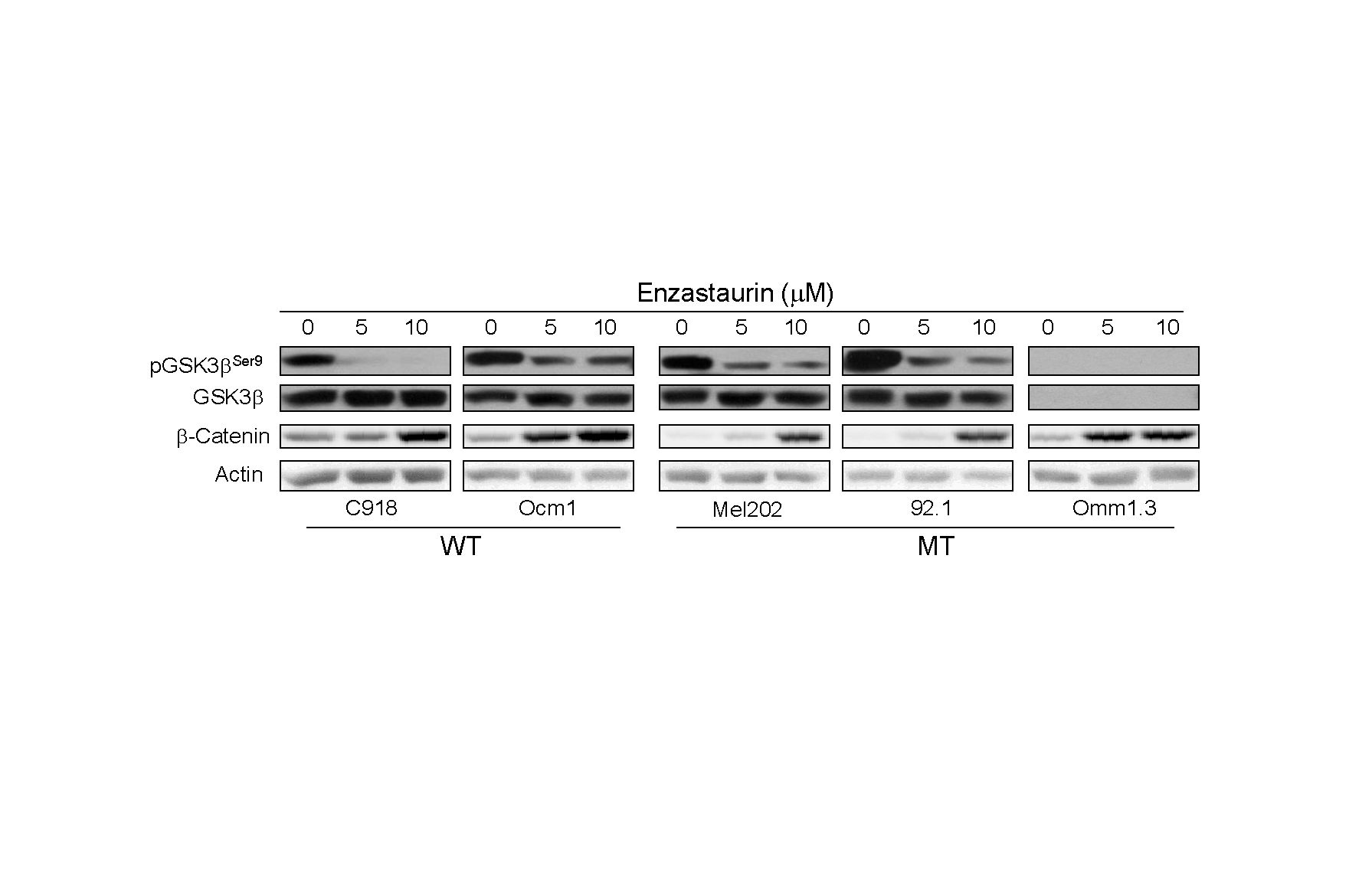

Supplement: Figure S3 — Effect of enzastaurin on GSK3β signaling in UM cells. Cells were treated with enzastaurin for 72 hours in the presence of 10% FBS. Note that the expression of GSK3β and pGSK3β Ser9 was under detectable levels of immunoblot in Omm1.3 cells. (TIF) [file pone.0029622.s003.tif]

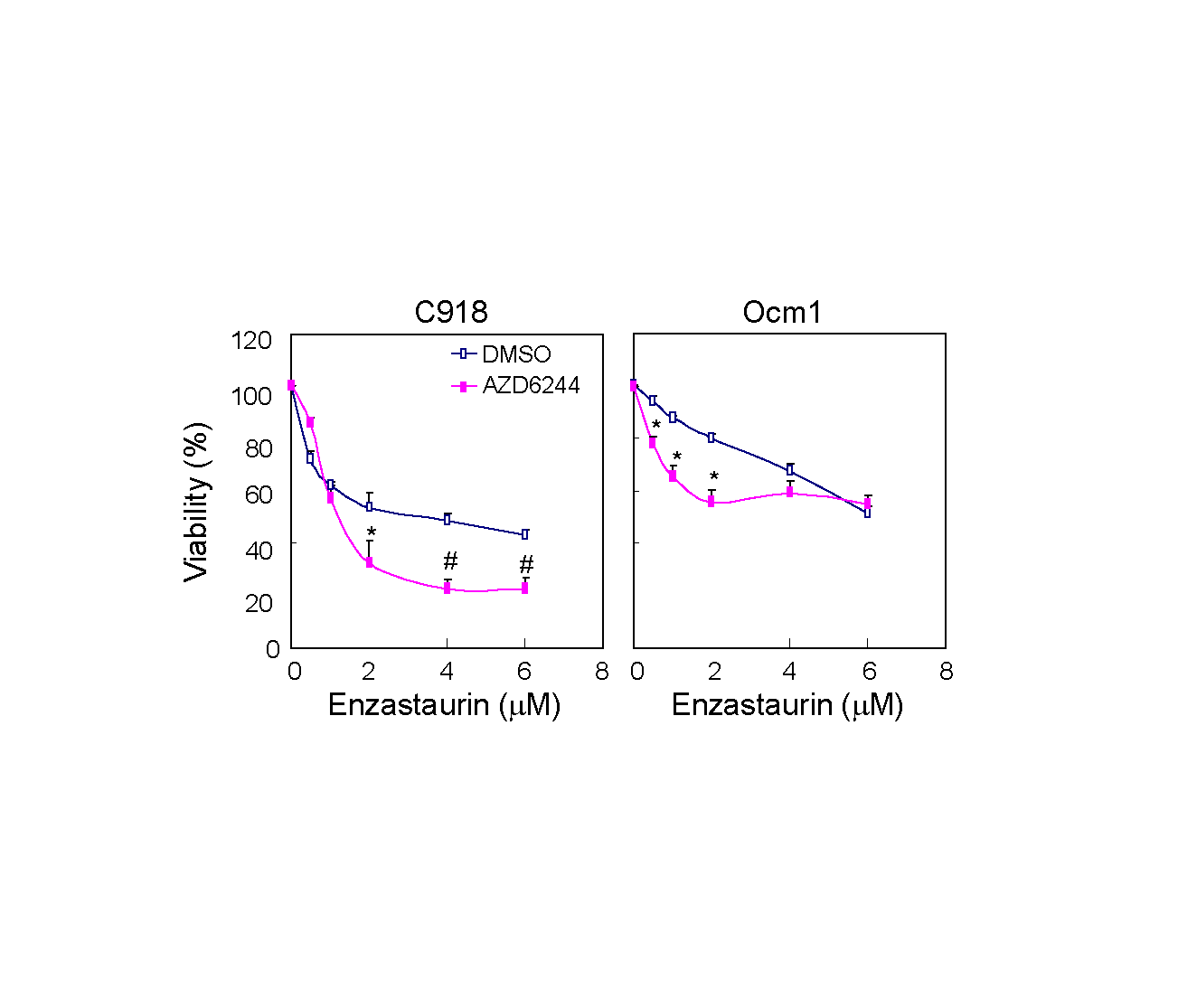

Supplement: Figure S4 — Effect of AZD6244 on the antiproliferative activity of enzastaurin. Assay was performed as described in Figure 1 in the absence or presence of 2 µM AZD6244. Results are presented as mean ± SD of percent viability from 2–3 independent experiments. * P<0.05, versus DMSO; # P<0.01, versus DMSO at the same dose of enzastaurin. (TIF) [file pone.0029622.s004.tif]

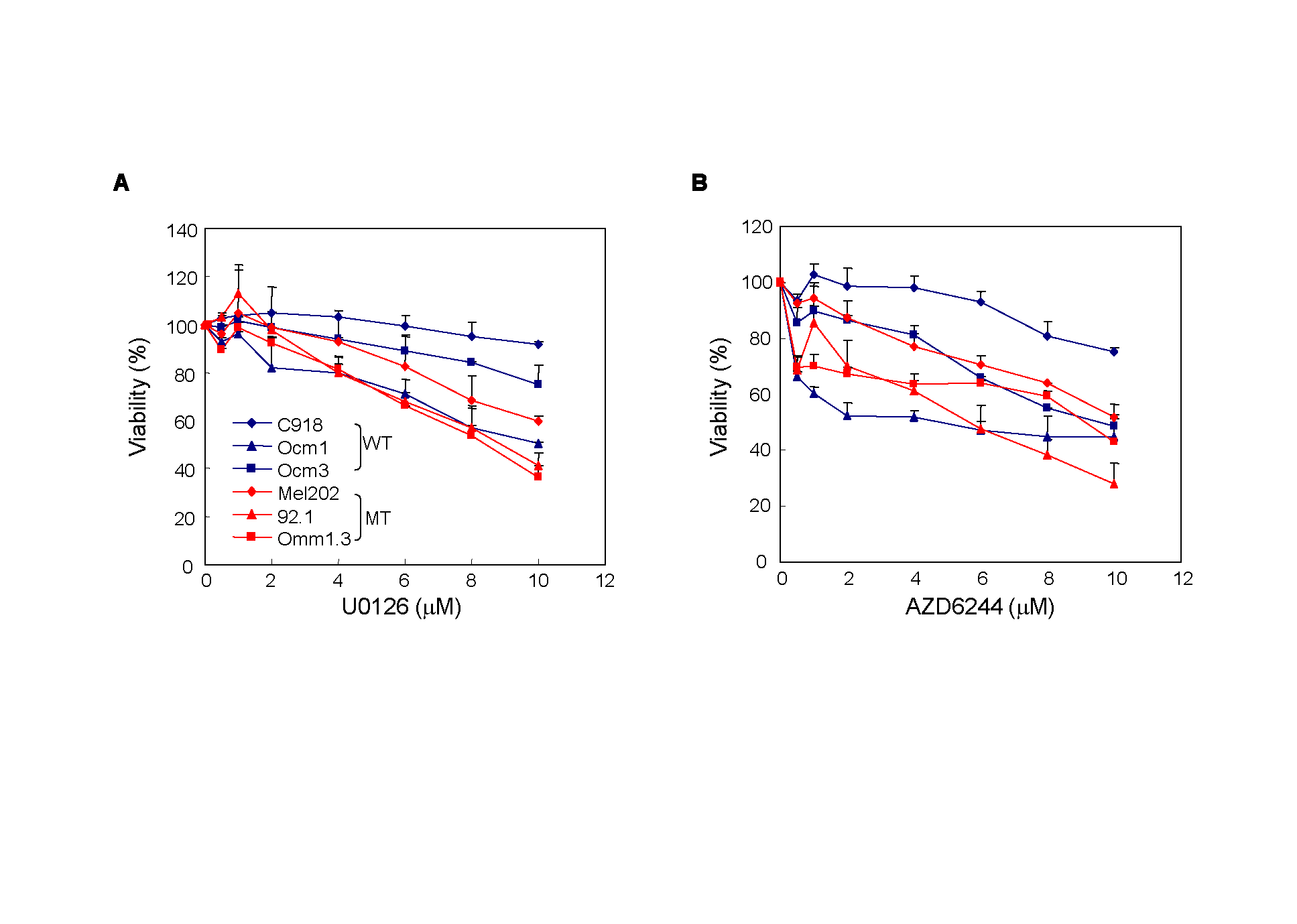

Supplement: Figure S5 — Effect of MEK inhibitors on UM cell viability. A, U0126. B, AZD6244. UM cells were treated with varying amount of U0126 or AZD6244 for 72 hours and subjected to MTS assay. Results are presented as mean ± SD of percent viability from 2 independent experiments. (TIF) [file pone.0029622.s005.tif]

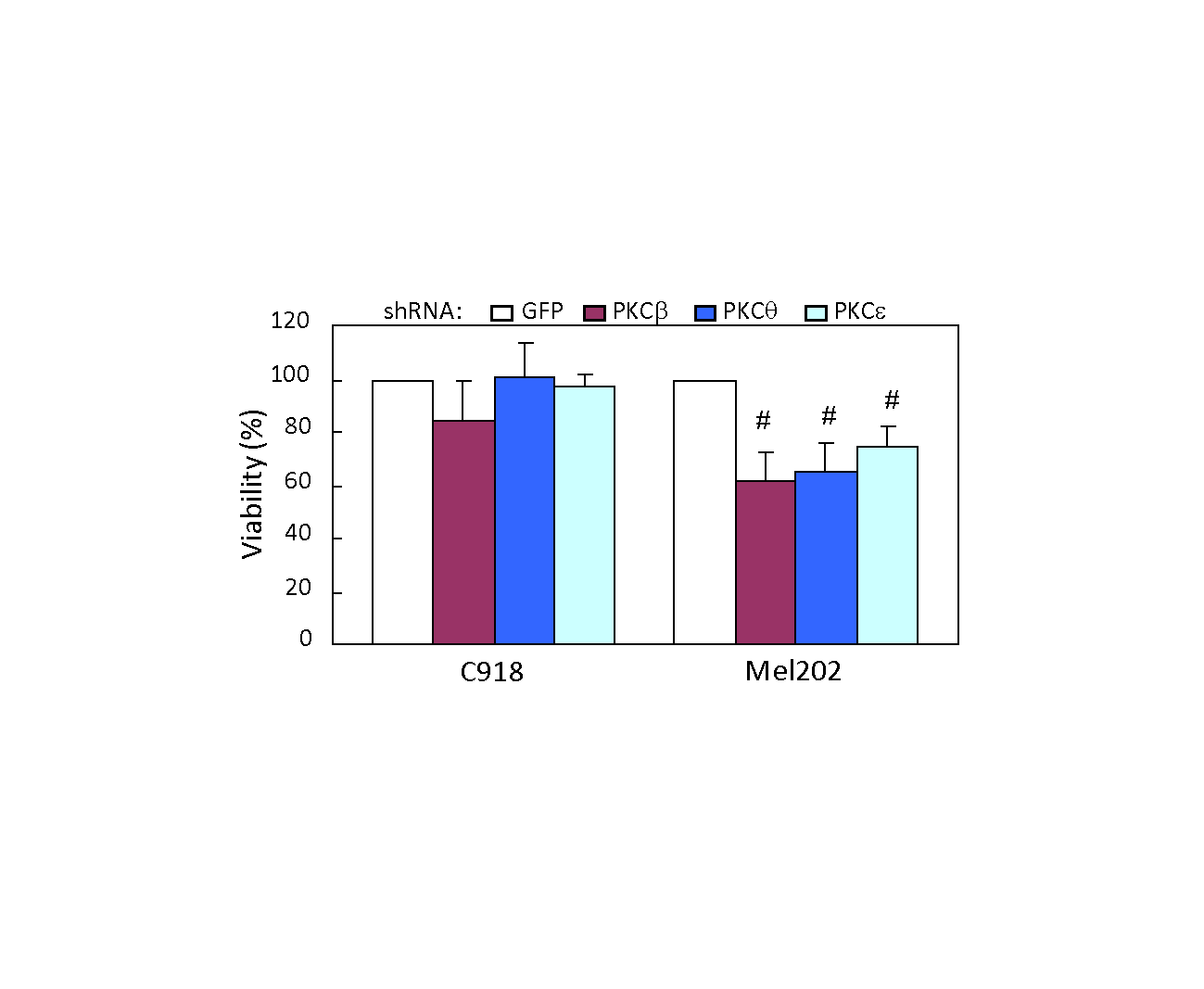

Supplement: Figure S6 — Effect of second shRNA for PKCβ, PKCε, and PKCθ on C918 and Mel202 cell viability. Experiments were performed as described in figure 8 . Results are presented as mean ± SD of percent viability from 3 independent experiments. # P<0.01 versus cells expressing GFP control shRNA. (TIF) [file pone.0029622.s006.tif]

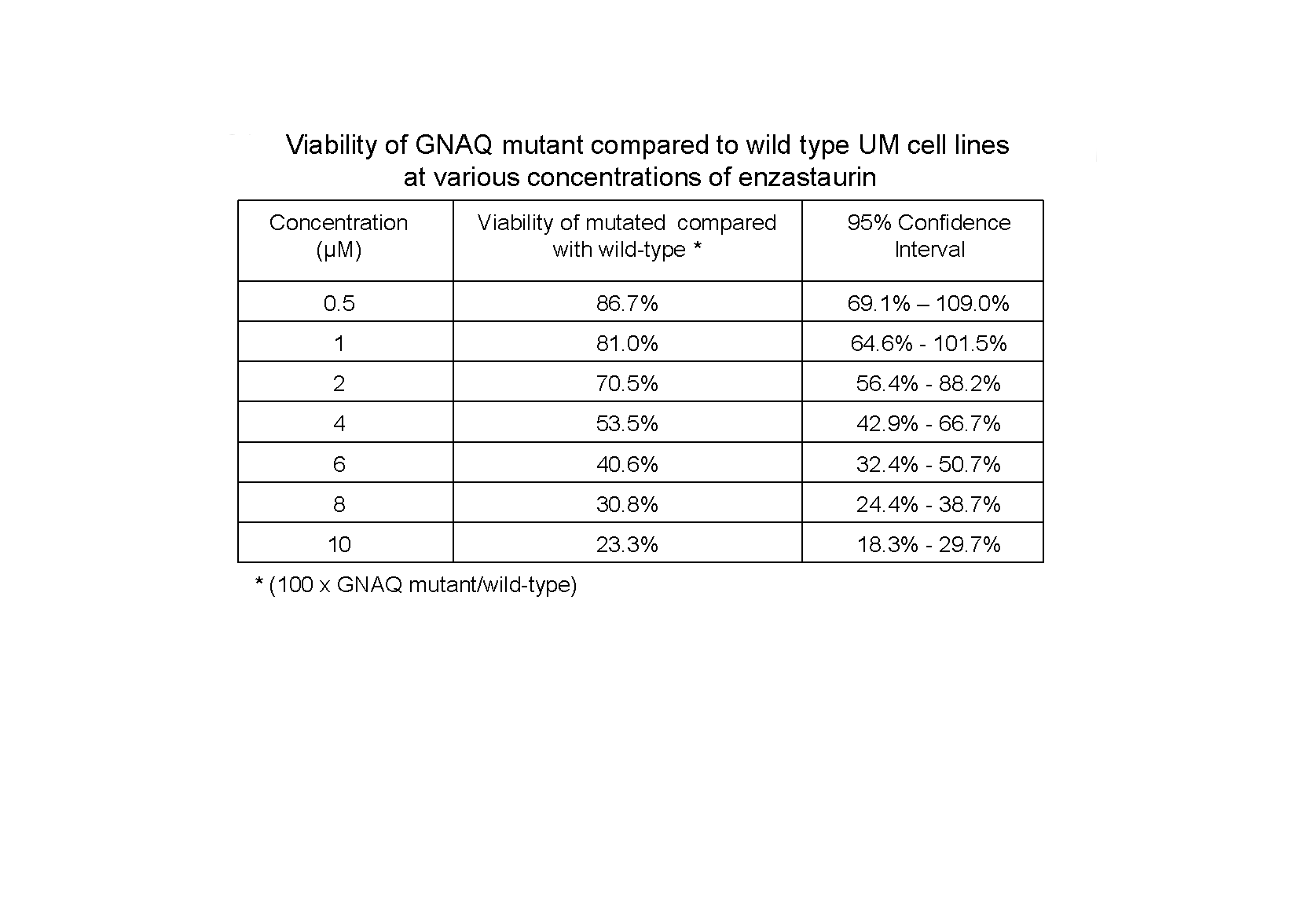

Supplement: Table S1 — Viability reduction rate of GNAQ mutant compared to wild type UM cell lines at various concentrations of enzastaurin. (TIF) [file pone.0029622.s007.tif]
